# Current and past depression as risk factors for incident type 2 diabetes mellitus and pre-diabetes in men and women: evidence from a longitudinal community cohort

**DOI:** 10.1186/s13098-024-01273-4

**Published:** 2024-02-01

**Authors:** Felix S. Wicke, Daniëlle Otten, Andreas Schulz, Philipp S. Wild, Karl J. Lackner, Thomas Münzel, Jochem König, Mareike Ernst, Jörg Wiltink, Iris Reiner, Jasmin Ghaemi Kerahrodi, Norbert Pfeiffer, Manfred E. Beutel

**Affiliations:** 1 Department of Psychosomatic Medicine and Psychotherapy, University Medical Center of the Johannes Gutenberg-University Mainz, Mainz, Germany; 2 Preventive Cardiology and Preventive Medicine, Department of Cardiology, University Medical Center of the Johannes Gutenberg-University Mainz, Mainz, Germany; 3 Center for Thrombosis and Hemostasis, University Medical Center, Johannes Gutenberg-University Mainz, Mainz, Germany; 4 German Center for Cardiovascular Research (DZHK), Partner Site Rhine-Main, Mainz, Germany; 5 Institute of Molecular Biology (IMB), Mainz, Germany; 6 Institute of Clinical Chemistry and Laboratory Medicine, University Medical Center of the Johannes Gutenberg-University Mainz, Mainz, Germany; 7 Center for Cardiology-Cardiology I, University Medical Center of the Johannes Gutenberg-University Mainz, Mainz, Germany; 8 Institute of Medical Biostatistics, Epidemiology and Informatics, University Medical Center of the Johannes Gutenberg-University Mainz, Mainz, Germany; 9 Department of Psychology, Psychotherapy and Psychoanalysis, Institute of Psychology, University of Klagenfurt, Klagenfurt am Wörthersee, Austria; 10 Faculty of Social Work, Darmstadt University of Applied Sciences, Darmstadt, Germany; 11 Department of Ophthalmology, University Medical Center, Johannes Gutenberg University Mainz, Mainz, Germany

**Keywords:** Diabetes, Type 2 diabetes, Prediabetes, Depression, Cohort study, Sex

## Abstract

**Background:**

Depression is associated with an increased risk for type 2 diabetes mellitus. However, depression may take different courses, and it is not fully understood how these affect the development of diabetes. It is further to be determined whether sex modifies the association between depression and type 2 diabetes.

**Methods:**

We analyzed data from the Gutenberg Health Study, a longitudinal and population-based cohort study (N = 15,010) in Germany. Depressive symptoms (measured by PHQ-9), history of depression, diabetes mellitus, and relevant covariates were assessed at baseline, and the outcomes of prediabetes and type 2 diabetes mellitus were evaluated 5 years later. Logistic regression was used to estimate odds ratios of incident prediabetes and type 2 diabetes mellitus, adjusting for potential confounders as identified in a Directed Acyclic Graph.

**Results:**

In the confounder adjusted model, current depression (PHQ-9 ≥ 10 at baseline; OR = 1.79, 95% CI = 1.11 to 2.74, p = 0.011), and persistent depression had a statistically significant (OR = 2.44, 95% CI = 1.62 to 3.54, p = 0.005) effect on incident type 2 diabetes mellitus. A history of depression without current depression had no statistically significant effect on type 2 diabetes (OR = 1.00, 95% CI = 0.68 to 1.43, p = 0.999). The effect of depression on incident diabetes did not differ significantly between women (OR = 2.02; 95% CI = 1.32 to 3.09) and men (OR = 2.16; 95% CI = 1.41 to 3.31; p-value for interaction on the multiplicative scale p = 0.832 and on the additive scale p = 0.149). Depression did not have a significant effect on incident prediabetes.

**Conclusion:**

This study shows how the history and trajectory of depression shape the risk for diabetes. This raises interesting questions on the cumulative effects of depression trajectories on diabetes and body metabolism in general. Depression can negatively affect physical health, contributing to increased morbidity and mortality in people with mental disorders.

**Supplementary Information:**

The online version contains supplementary material available at 10.1186/s13098-024-01273-4.

## Background

Depression is one of the most frequent and harmful mental disorders. It has an estimated lifetime risk of 15–25% and often takes a chronic course [1]. Depression is associated with elevated morbidity and mortality [1]. In aging societies, chronic diseases, such as type 2 diabetes mellitus, have become the leading causes of death [2]. Diabetes mellitus and depression are often comorbid [3]. The relationship between type 2 diabetes and depression is considered chiefly bidirectional [4–7]. The prevalence of depression is higher in people with type 2 diabetes than in those without diabetes [8, 9]. Reviews reveal depression to be associated with a 37% [10] to 60% increased risk of type 2 diabetes [4].

While depression has gained strong consideration as important risk factor in cardiovascular diseases [11], which lead to a growing awareness of the psychosomatic associations of depression and the importance of its treatment not only for mental, but also for physical health, we believe that depression did not receive the same amount of attention in regard to diabetes. A deepened understand of the association, however, can be of high clinical relevance for both diabetologists and mental health professionals.

Different mechanisms have been suggested to explain how depression contributes to the development of type 2 diabetes [12, 13]. They can be roughly divided into (behavioral) lifestyle factors and biological factors.

Among lifestyle factors are physical activity, which is commonly reduced in depression. Physical inactivity contributes to the development of obesity, which is among the major risk factors of type 2 diabetes [14]; however, physical activity has beneficial effects on diabetes independent of body weight, e.g. by changing muscle metabolism of glucose. Additionally, depression can impair adherence to healthy diets and can increase appetite, both of which can increase the risk of type 2 diabetes. Smoking is another lifestyle factor of consideration, as smoking increases the risk of type 2 diabetes. Depression also likely causes or worsens smoking in many patients, although the evidence on this is inconsistent [15].

Hormonal changes as explanatory link between depression and type 2 diabetes are based on the idea that depression is associated with, or causes a chronic stress response with corresponding release of stress-related hormones via the hypothalamic–pituitary–adrenal (HPA) axis. Among these are catecholamines and glucocorticoids, both of which increase blood glucose [12, 13]. In addition to hormonal changes, inflammation possibly links depression to type 2 diabetes, as depression is associated to inflammatory responses and this can increase risk of type 2 diabetes. C-reactive protein and interleukin 6, for example, predicted increased risk for depression and type 2 diabetes [13]. Some antidepressants are associated with weight gain and increased risk of type 2 diabetes [13, 16, 17] and we consider them as a further potential mediator of the association.

**Fig. 1:**
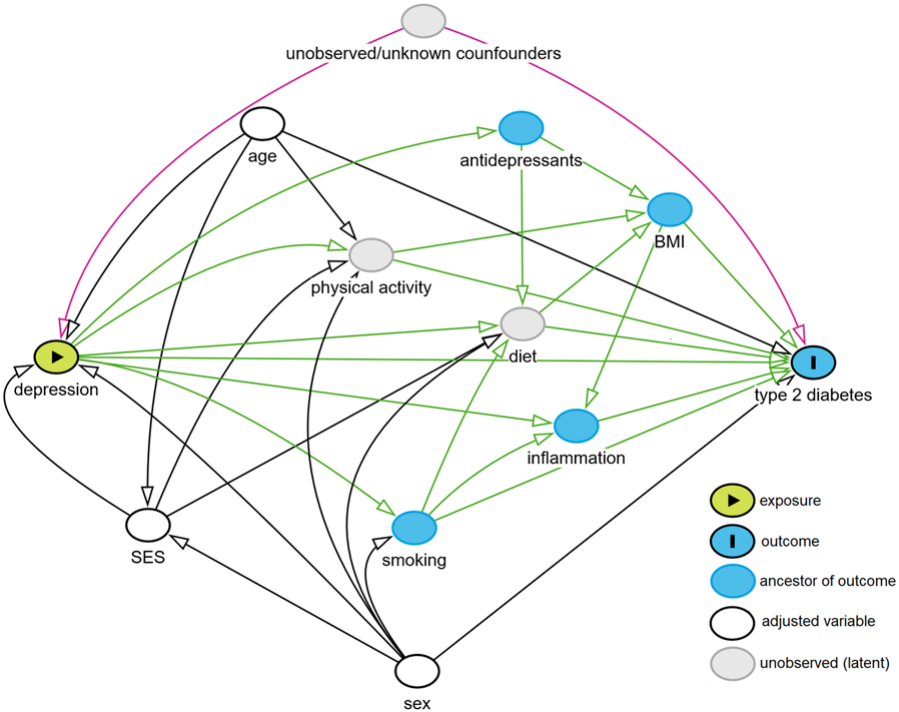
Directed acyclic graph (DAG) of the proposed causal model between depression and type 2 diabetes. An interactive version of the DAG can be found here: https://dagitty.net/mGmFkw2Bg (correct adjustment can be checked by setting “unobserved/unknown confounders” to “adjusted”). *SES* socioeconomic status, *BMI* body mass index

Figure 1 shows a directed acyclic graph (DAG, interactive version: https://dagitty.net/dags.html?id=nN2ky3og) of our proposed causal model explaining how depression could lead to the development of type 2 diabetes. As the association of depression and type 2 diabetes could be confounded by third factors in this observational study, we also tried to include the most relevant confounders. These are primarily age, sex and socioeconomic status. The role of sex is discussed further below. Other possible confounders are prematurity at birth, adverse childhood events and mental stress other than depression (we summarized them as “unknown and unobserved confounders” in the DAG).

In the DAG we tried to depict what we consider to be the most relevant associations explaining the link between depression and diabetes. In addition, we want to elaborate here on the role of prediabetes and of the trajectory of depression.

Prediabetes is an intermediate stage between normal glycemia and diabetes [18] and associated with a four- to 12-fold increased risk of developing type 2 diabetes [19, 20]. We assume that prediabetes is caused by the same etiological factors as diabetes itself, albeit with differences in the severity of the pathogenic processes (we therefore did not include prediabetes in the DAG). If depression was a risk factor for prediabetes, preventive strategies that addressed this could be effective to reduce incidence of type 2 diabetes, as both depression and prediabetes can be detected rather easily.

Duration and severity of depression are most likely important factors to explain how depression contributes to the pathogenesis of type 2 diabetes. For example, the duration of obesity is a relevant factor in the pathogenesis of type 2 diabetes, independent of baseline BMI [21–24]. However, most studies consider depression as a singular factor [4, 9].

Concerning sex and/or gender differences, the prevalence of depression was higher in women with type 2 diabetes [9], which reflects the epidemiology of depression in the general population. Furthermore, sex-specific findings concerning the impact of depressive symptoms on diabetes are conflicting, indicating either no effects or only an effect for women [25]. In a previous study using data from the Gutenberg Health Study (GHS), the predictive effect of current depressive symptoms on the incidence of type 2 diabetes 5 years later disappeared for men after metabolic (BMI, dyslipidemia, obesity, blood glucose, and hypertension) and lifestyle factors (physical activity and smoking) were statistically taken into account. For women, the effect remained significant. For both women and men, BMI, blood glucose, and hypertension increased the probability of diabetes mellitus, and for men, dyslipidemia and physical inactivity increased the probability of diabetes [26]. Of note, sex/gender is a potential confounder of the association between depression and type 2 diabetes, but it could also be a moderator, as there are likely differences in physiology and metabolism between women and men, that could explain the observed differences in diabetes incidence.

### Objectives

To expand the knowledge on associations between depression and type 2 diabetes, we studied the effects of depression on the incidence of prediabetes and type 2 diabetes in a longitudinal design. As a knowledge gap exists about the effect of the trajectory and history of depression on incident diabetes, we explore not only the impact of a current depressive episode but also the effect of past depression only and of persistent depression on the incidence of prediabetes and type 2 diabetes mellitus. In addition, due to the indeterminate findings on the influence of sex, we studied whether sex modifies the association between depression and incident type 2 diabetes.

In summary, the main goals of this study are to examine (1) whether depression (prior episode, current episode, and persistent depression) affects the incidence of type 2 diabetes, (2) whether sex modifies the association between depression and type 2 diabetes, and (3) whether depression (and its history) is an additional risk factor for prediabetes.

## Methods

### Study design

The Gutenberg Health Study (GHS) is a population-representative, prospective, single-center cohort study in the Rhine-Main-Region, Germany [27]. The study protocol and documents were approved by the ethics committee of the Medical Chamber of Rhineland-Palatinate (reference no. 837.020.07; original vote: 22.3.2007, latest update: 20.10.2015) and the local data safety commissioner. All study investigations were conducted per the Declaration of Helsinki and principles outlined in recommendations for Good Clinical Practice and Epidemiological Practice. Presentation in this manuscript is oriented on the STROBE checklist for cohort studies [28]. Before enrollment, participants signed written informed consent. The GHS is not registered in a public trial register.

### Setting and participants

The sample was drawn randomly from the local population register of the city Mainz and the district Mainz-Bingen, stratified 1:1 for sex and residence and in equal strata for decades of age. The inclusion criterion was age 35 to 74 years. Exclusion criteria were insufficient knowledge of the German language and psychological or physical impairment prohibitive of participation in tests and interviews. The response proportion (defined as the recruitment efficacy proportion, i.e., the number of persons who participated in the baseline examination divided by the sum of the number of persons who participated in the baseline examination plus those who refused and those who were not contactable) was 55.5%.

Participants were examined between 2007 and 2012 in a standardized 5-h study center visit and again 5 years later at a follow-up visit. For the present analysis, we excluded participants with a diagnosis of diabetes other than type 2 at baseline.

### Variables

Participants completed self-report questionnaires, including standardized psychometric measures. During the 5-h study-center visit, computer‐assisted interviews, anthropometric measures, and routine laboratory assessments were conducted in a standardized manner to assess cardiovascular risk factors, disease history (participants were asked about physician-diagnosed diseases during the computer-assisted interviews), and humoral biomarkers of glucose metabolism. Medication history was derived from medical records and personal reports and categorized according to the Anatomical Therapeutic Chemical Classification System.

The Depression module of the Patient Health Questionnaire (PHQ-9), a widely used self-report instrument, measured depressive symptoms at baseline. A sum score of 10 or more was used as the cutoff to define current depression [29, 30]. The term current depression refers in our study to the presence of depression in the baseline assessment. The PHQ-9 is a reliable measure for depression; within the present sample, internal consistency for the PHQ-9 was good, with McDonald’s omega of 0.96 [31]. History of depression was assessed in the computer-assisted interview by asking participants about a prior definite diagnosis of depression by a physician. We classified participants into four categories: (1) no depression (neither prior nor current depression), (2) a likely episode of depression (current depression as defined by a PHQ-9 score of at least 10 points, but no prior depression), (3) history of depression (prior, but no current depression), and (4) persistent depression (current and prior depression).

Participants were classified as having diabetes mellitus if they reported taking antidiabetic medication and/or reported a previous diagnosis of type 2 diabetes mellitus and/or had an HbA1c ≥ 6.5% (47.5 mmol/mol). Prediabetes was present if participants had an HbA1c of 5.7–6.4% (38.8–46.5 mmol/mol) following the American Diabetes Association criteria [32]. Concentrations of HbA1c (and further clinical chemistry parameters) were determined under standardized conditions within the daily clinical routine diagnostics at the Institute of Clinical Chemistry and Laboratory Medicine of the University Medical Centre Mainz. HbA1c was measured by high-performance liquid chromatography (Bio-Rad Laboratories, Hercules, California, USA).

Education, occupation, and household income were each categorized into a nominal scale with 1 indicating the lowest and 7 meaning the highest status category and then combined into a single measure of socioeconomic status (SES), as described by Lampert et al. [33]. Body mass index (BMI) was calculated by dividing body weight in kilograms by squared body height in meters. Pack‐years of smoking were calculated as the number of cigarettes smoked per day divided by 20 (a pack) and multiplied by the number of years smoked. C-reactive protein (CRP) concentration was measured in heparin-plasma after venous blood sampling by a high-sensitivity latex enhanced immunoturbidimetric assay (Abbott Laboratories, Abbott Park, IL; limit of detection: 0.2 mg/L).

Antidepressants were categorized into those with and those without known effects on body weight [34], assuming that this reflects potential diabetogenic properties.

### Bias

The Gutenberg-Health-Study used population-based sampling to reduce selection bias and a highly standardized assessment procedure with professional study personnel that was independent of specific interest in research questions to reduce measurement related biases.

### Study size

The size of the cohort was initially planned for the assessment of cardiovascular outcomes, which was unrelated to the aims of this study on depression and diabetes. For this study, we therefore used all available participant data to maximize power.

### Statistical methods

Descriptive analyses were performed as absolute and relative proportions for categorical data and means and standard deviations for continuous variables.

The main outcomes were incident prediabetes and incident type 2 diabetes mellitus. To study these outcomes, two analysis samples were created: the first included all participants with neither prediabetes nor diabetes at baseline and was used to study incident prediabetes; the second included all participants without diabetes at baseline and was used to study incident type 2 diabetes. Outcome status was assessed 5 years after the baseline examination using the case definitions of prediabetes and diabetes described above.

Multiple logistic regression models were used to estimate the relative odds (odds ratio, OR) of incident prediabetes and incident type 2 diabetes, dependent on depression status (no depression, current depression, history of depression, and persistent depression). In Model 1 we did only basic adjustment for age and sex.

Based on the DAG (Fig. 1) we selected adjustment sets for multiple regression analysis. For estimation of the *total effect* (Model 2) of depression on type 2 diabetes, the sufficient adjustment set included age, sex and SES (and unknown/unobserved confounders, which cannot be adjusted for). Under the assumption of no bias or confounding, the *total effect* is the true estimate of depression’s causal effect on type 2 diabetes incidence. In addition, we tried to estimate the *direct effect* (Model 3) of depression on type 2 diabetes, i.e. the effect that is not accounted for by the known and measured mediators. Model 3, therefore, was adjusted for age, sex, SES, BMI, smoking, CRP (as measure of inflammation), and antidepressant medication.

Due to severe skewness, both pack-years of smoking and CRP were log-transformed, and never smokers (pack-years = 0) were included as separate covariate.

To assess sex as a potential modifier of the relation between depression and type 2 diabetes, odds ratios of incident type 2 diabetes stratified by sex and depression status at baseline (PHQ-9 < 10 and PHQ-9 ≥ 10) were calculated. To test statistical significance of any differences in odds ratios, the interaction term (multiplicative scale) and the relative excess risk due to interaction (RERI) were calculated, as recommended by Knol and VaderWeele [35].

Statistical analyses were computed with R version 4.2.2 [36] utilizing the packages psych [37], dplyr [38] and interactionR [39].

## Results

### Participants and baseline characteristics

**Fig. 2:**
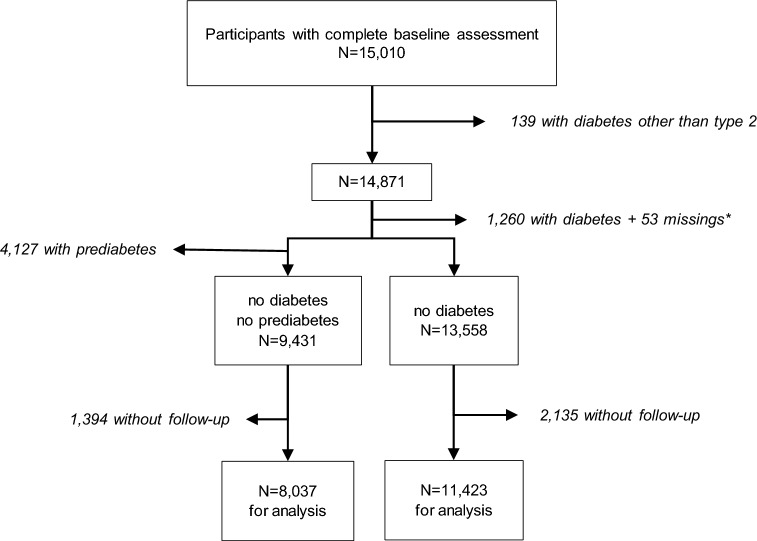
Flow diagram showing the derivation of analysis samples for incident cases of diabetes resp. prediabetes. *Missing diabetes status at baseline

Out of 15,010 participants with complete baseline data, 139 (0.93%) had a diagnosis of diabetes other than type 2 and were excluded from this analysis (compare Fig. 2). The average age of the remaining 14,871 participants was 55 years, and 49.6% were women. At baseline, 1260 (8.5%) participants fulfilled the criteria for type 2 diabetes, and 5329 (36.0%) fulfilled the criteria for prediabetes. For the study of incident diabetes, all 11,423 participants without diabetes at baseline and with 5-year follow-up data available were selected. For the study of incident prediabetes, all 8037 participants without pre-diabetes or diabetes at baseline and with 5-year follow-up data available were selected. Because the main focus of this study is on incident diabetes, Table 1 shows the detailed baseline characteristics of the 11,423 participants used for the study of incident diabetes. The detailed characteristics of the total sample and the prediabetes sample are shown in Additional file 1: Tables S1 and S2. Numbers of missing values for relevant variables are reported in Additional file 1: Table S3.

**Table 1:** Baseline characteristics of participants without diabetes at baseline

	Overall (n = 11,432)	Men (n = 5731)	Women (n = 5692)
Age (mean (SD))	53.84 (10.80)	53.95 (10.86)	53.74 (10.74)
Women (%)	5692 (49.8)		
SES (mean (SD))	13.37 (4.39)	14.14 (4.50)	12.59 (4.13)
PHQ-9 (mean (SD))	3.98 (3.46)	3.54 (3.30)	4.42 (3.57)
Current depression, PHQ-9 ≥ 10 (%)	787 (7.0)	310 (5.5)	477 (8.5)
Depression status^a^			
No depression (%)	9609 (85.1)	5047 (89.2)	4562 (81.0)
HD (%)	898 (8.0)	304 (5.4)	594 (10.5)
CD (%)	405 (3.6)	188 (3.3)	217 (3.9)
PD (%)	378 (3.3)	120 (2.1)	258 (4.6)
BMI (mean (SD))	26.84 (4.60)	27.40 (3.97)	26.27 (5.11)
Packyears of smoking (mean (SD))	4.26 (10.31)	5.09 (11.66)	3.47 (8.76)
Prediabetes^c^ (%)	3386 (29.6)	1712 (29.9)	1674 (29.4)
HbA1c (mean (SD))	5.42 (0.43)	5.43 (0.42)	5.42 (0.44)
CRP (mean (SD))	2.63 (4.73)	2.42 (4.15)	2.84 (5.24)
Antidepressant with weight effect (%)	147 (1.3)	41 (0.7)	106 (1.9)
Weight-neutral antidepressant (%)	318 (2.8)	109 (1.9)	209 (3.7)
Diabetes^b^ at F/U (%)	455 (4.0)	266 (4.7)	189 (3.3)
Prediabetes^c^ at F/U (%)	3782 (33.2)	1847 (32.3)	1935 (34.2)

^a^Classification of depression status: current depression is defined by PHQ-9 ≥ 10, prior depression is based on patient’s report of a prior diagnosis of depression (history of depression)

^b^Diabetes defined by intake of antidiabetic medication and/or previous diagnosis of type 2 diabetes mellitus and/or HbA1c ≥ 6.5%

^c^Pre-diabetes defined by HbA1c 5.7–6.4% (following the American Diabetes Association criteria)

*SES* socioeconomic status, *PHQ-9* Patient Health Questionnaire—Depression Module, *HD* history of depression (without current depression as defined by PHQ-9 ≥ 19), *CD* current depression (PHQ-9 ≥ 10, without history of depression), *PD* persistent depression (history of depression and current depression as defined by PHQ-9 ≥ 10), *BMI* body mass index, *HbA1c* glycated hemoglobin concentration, *CRP* C-reactive protein

### Outcome data

After 5 years, 1600 (19.9%) participants of 8037 without diabetes or prediabetes at baseline fulfilled prediabetes criteria at follow-up. Notably, 1191 (28.9%) of the 4127 participants with prediabetes at baseline did not fulfill the follow-up requirements (i.e., glucose metabolism improvement). Type 2 diabetes developed in 455 (4.0%) of 11,423 participants without prediabetes or diabetes at baseline.

### Main results

The effects of depression (and its trajectory) on the incidence of prediabetes and type 2 diabetes mellitus are reported in Table 2. Our fist aim was to estimate the effect of depression on incidence of type 2 diabetes. When adjusting for age and sex only (Model 1), current depression as defined by a PHQ-9 ≥ 10 point (OR = 1.84, 95% CI = 1.14 to 2.81) and persistent depression (OR = 2.61, 95% CI = 1.74 to 3.79) were statistically significant predictors of type 2 diabetes, but only having a history of depression was not. The confounder-adjusted estimates (total effect, Model 2) of current depression showed an OR of 1.79 (95% CI = 1.11 to 2.74) and of persistent depression an OR of 2.44 (95% CI = 1.62 to 3.54). Inclusion of the proposed mediators in the adjustment set reduced the estimates to an OR of 1.41 (95% CI = 0.85 to 2.24) for current depression and to an OR of 2.02 (95% CI = 1.25 to 3.14) for persistent depression. More detailed results, including coefficients of covariates, are shown in Additional file 1: Table S4.

**Table 2:** Results of logistic regression analysis: relative odds of incident prediabetes and type 2 diabetes mellitus, predicted by depression status (history of depression, current depression, and persistent depression; with no depression as reference category)

	Prediabetes^a^				Diabetes^b^			
	OR	95% CI		p value	OR	95% CI		p value
	Model 1 (n = 7922)				Model 1 (n = 11,243)			
HD	0.97	0.79	1.19	0.778	1.04	0.71	1.47	0.848
CD	1.11	0.81	1.48	0.511	1.84	1.14	2.81	0.008
PD	1.19	0.86	1.61	0.276	2.61	1.74	3.79	< 0.001

	Model 2 (n = 7895)				Model 2 (n = 11,203)			
HD	0.95	0.77	1.16	0.628	1.00	0.68	1.43	0.999
CD	1.06	0.78	1.43	0.688	1.79	1.11	2.74	0.011
PD	1.15	0.83	1.55	0.391	2.44	1.62	3.54	< 0.001

	Model 3 (n = 7532)				Model 3 (n = 10.706)			
HD	0.85	0.67	1.06	0.152	0.90	0.59	1.34	0.627
CD	0.96	0.70	1.31	0.819	1.41	0.85	2.24	0.162
PD	0.95	0.66	1.33	0.752	2.02	1.25	3.14	0.003

Model 1 adjusted for age and sex. Model 2 adjusted for age, sex and socioeconomic status

Model 3 adjusted for age, sex, SES, BMI, CRP, pack-years, antidepressants with weight effects, and antidepressants without weight effects

*OR* odds ratio, *95% CI* 95% confidence interval, *HD* history of depression (without current depression as defined by PHQ-9 ≥ 19), *CD* current depression (PHQ-9 ≥ 10, without history of depression), *PD* persistent depression (history of depression and current depression as defined by PHQ-9 ≥ 10), *SES* socioeconomic status, *BMI* body mass index, *CRP* C-reactive protein

^a^Prediabetes defined by HbA1c 5.7–6.4% (following the American Diabetes Association criteria)

^b^Diabetes defined by intake of antidiabetic medication and/or previous diagnosis of type 2 diabetes mellitus and/or HbA1c ≥ 6.5%

The second aim of this study was to assess whether sex modifies the effect of depression on diabetes incidence. For this analysis, we used confounder-adjusted (total effect) estimates, adjusted for age, sex and SES, as derived from the DAG. Results are reported in Table 3. Compared to women without depression, men with depression had the highest relative odds of developing type 2 diabetes (OR = 3.54; 95% CI = 2.29 to 5.49). The effect of depression on incident diabetes, however, did not differ significantly between women (OR = 2.02; 95% CI = 1.32 to 3.09) and men (OR = 2.16; 95% CI = 1.41 to 3.31). Tests for interaction were not statistically significant on the multiplicative (interaction term = 1.07; 95% CI = 0.59 to 1.95) or additive scale (RERI = 0.88; 95% CI = − 0.78 to 2.54).

**Table 3:** Effect of sex on the association between depression and incident type 2 diabetes mellitus (n = 11,203)

	Depression		Effect of depression by sex
	No (PHQ-9 < 10)	Yes (PHQ-9 ≥ 10)	
	OR [95% CI]	OR [95% CI]	OR [95% CI]
Women	1 (Reference)	2.02 [1.32, 3.09]	2.02 [1.32, 3.09]
Men	1.64 [1.33, 2.03]	3.54 [2.29, 5.49]	2.16 [1.41, 3.31]
Multiplicative scale (interaction term): 1.07 [0.59, 1.95], p = 0.832			
Additive scale (RERI): 0.88 [− 0.78, 2.54], p = 0.149			

Model adjusted for age, sex and socioeconomic status

*PHQ-9* Patient Health Questionnaire—Depression Module, *OR* odds ratio, *CI* confidence interval, *RERI* relative excess risk due to interaction

The third aim was to study whether the observations of depression’s effect on diabetes incidence could also be shown for prediabetes. Results are shown in Table 2. The Models did not show any statistically significant effect of depression (history, current or persistent depression) on prediabetes.

## Discussion

In this study, we examined how past and current depression predict the incidence of pre-diabetes and type 2 diabetes mellitus over 5 years within a population-representative adult prospective cohort study in Germany. Furthermore, we studied the modifying effect of sex.

Here, we summarize the study’s key results, which we discuss individually in further detail below. First, our results support previous research that established an association of depression with incident diabetes mellitus. Notably, persistent depression emerged as a more potent risk factor for incident diabetes mellitus than baseline depression (as defined by PHQ-9 ≥ 10). Second, sex was not a relevant modifier of the effect of depression on type 2 diabetes incidence. Third, there were no demonstrable effects of an current depression or persistent depression on later prediabetes.

The findings of this study contribute to the existing research on the association between depression and type 2 diabetes mellitus by demonstrating the importance of acknowledging the history and trajectory of depression beyond assessments of depression at a single time point. A history of depression can reflect the duration of depression, the severity of depression, or both, and the results are in line with the idea that causal mechanisms linking depression and diabetes have more significant effects if depression is more severe and if they work over more extended periods (e.g., as cumulative exposure). The results further support evidence pointing to significant differences between chronic and nonchronic depression [40]. This could apply to psychophysiological factors, such as cumulative stress and allostatic load resulting in regular glucocorticoid production and diabetogenesis [13, 41], or to lifestyle factors, such as depressive lack of energy and inactivity and increases in appetite [42] or smoking.

The effect of depression on incident diabetes decreased after including mediators in the model. There are several possible explanations for this observation: (1) our model does not include all relevant mediators. Examples are diet and physical activity, at least for possible effects they have beyond what is explained by obesity (BMI). As discussed in the introduction, physical activity can influence muscle metabolism beneficially, independent of lipid metabolism. We could also not include neuro-hormonal mediators like glucocorticoid measures. (2) The measures of the mediators we included could by measured imperfectly or reflect only partially the involved pathogenic mechanisms. For example, we included CRP as a measure of inflammation, however, there are many other inflammatory mechanisms that could be diabetogenic, e.g. interleukin-6. (3) Residual confound by unknown or unobserved variables cannot be ruled out definitely.

Many, but not all, previous studies have demonstrated the effect of depression on type 2 diabetes incidence. Our findings support the results of systematic reviews that found such an effect [4, 9]. The inclusion of mediators in statistical models could mask some of the causal effects of depression on diabetes incidence and could explain why some studies do not find significant effects of depression on diabetes [26, 43]. We hope that the causal model we developed (see Fig. 1) will facilitate further research on the mechanisms linking depression with diabetes mellitus.

In this study, we did not find significant differences between women and men regarding the effect of depression on diabetes. This is in line with the results from the meta-analysis by 4 [4], where the pooled relative risk was 1.26 (95% CI 0.95–1.67) for women and 1.57 (95% CI 1.24–1.99).

The observation that there were no effects of depression on incident prediabetes is notable. One explanation could be that the HbA1c range of prediabetes reflects a very heterogeneous population, supported by its high prevalence and a high proportion of remission. The type 2 diabetes population, in contrast, could represent a more homogeneous group characterized by shared pathological mechanisms of carbohydrate metabolism with all its associated consequences. A meta-analysis by 44 [44] found a small association between depression and prediabetes with a pooled odds ratio of 1.11 (95% CI 1.03–1.19). Thus, further research needs to elucidate the potential influence of depression on prediabetes and the underlying biological mechanisms.

### Implications for clinical practice

In light of our results and of the many studies that demonstrated an association of depression and incident diabetes, we suggest, (1) that mental health professionals should assess (by themselves of recommend to e.g. the primary care physician) basic physical health status und risk factors like blood pressure, BMI, physical activity and nutrition and (psychological and social) barriers to the adoption of a healthy life-style; (2) that diabetologists should inform themselves on the mental health status of their patients with diabetes or at risk of diabetes; and (3) that clinicians should ask about the history of depression.

### Strengths and limitations

Unlike most previous studies, we explicitly account for persistent depression and the symptom score at the most recent measurement point. We cannot determine if the course of depression was indeed persistent or recurrent. However, it seems worthwhile to study the effect of different trajectories of depressive symptoms in future studies. The gold standard for assessment of depression and its trajectory, would be a detailed and structured clinical interview administered by a mental health professional. The PHQ-9 was developed to reflect the criteria of the diagnostic and statistical manual, but of course remains an imperfect measure of depression.

A particular strength of the study is its large and population-based sample with detailed assessment, which allowed us to consider the most important covariates. This is the most extensive study investigating the link between depression and diabetes with HbA1c-based laboratory assessment.

Few studies have specifically examined effect modification by sex with statistical tests. While we did not find evidence for effect modification of sex, this does not exclude sex differences due to a possible lack of statistical power.

## Conclusions

Our results emphasize the importance of examining a history of depression and the current episode of depression in testing associations between depression and type 2 diabetes. This raises interesting questions on the cumulative effects of depression trajectories on diabetes and the body’s metabolism in general. The study supports previous results that show how depression can negatively affect physical health status, which contributes to increased morbidity and mortality in mentally ill individuals. For clinical practice, mental and physical health interrelations should be considered and addressed, with targeted prevention and intervention efforts, e.g., support in lifestyle changes.

### Supplementary Information

**Additional file 1: Table S1.** Baseline characteristics of participants at baseline. **Table S2.** Baseline characteristics of participants without prediabetes and diabetes at baseline. **Table S3.** Missings (N = 14,871 at baseline). **Table S4.** Detailed results of logistic regression analysis: relative odds of incident pre-diabetes and type 2 diabetes mellitus, predicted by depression status (current depression, prior depression, or both) with odds ratios of covariates.

## Data Availability

Written informed consent from GHS study participants does not allow public access to the data. Access to the data in the local database is possible at any time upon request, according to the ethics vote. This concept was developed with the local data protection officer and the ethics committee (local ethics committee of the Rhineland-Palatinate Medical Association, Germany). Interested scientists can make their requests to the Gutenberg Health Study Steering Committee (e-mail: info@ghs-mainz.de).
