# Supplementary material for: Current and past depression as risk factors for incident type 2 diabetes mellitus and pre-diabetes in men and women: evidence from a longitudinal community cohort
Source: Diabetol Metab Syndr. 2024 Feb 1;16:34. doi: 10.1186/s13098-024-01273-4 (PMC10832228; doi:10.1186/s13098-024-01273-4)
Supplement: Supplementary file 1 — Additional file 1: Table S1. Baseline characteristics of participants at baseline. Table S2. Baseline characteristics of participants without prediabetes and diabetes at baseline. Table S3. Missings (N = 14,871 at baseline). Table S4. Detailed results of logistic regression analysis: relative odds of incident pre-diabetes and type 2 diabetes mellitus, predicted by depression status (current depression, prior depression, or both) with odds ratios of covariates. [file 13098_2024_1273_MOESM1_ESM.docx]

**Supplement to Wicke et al.: Current and past depression as risk factors for incident type 2 diabetes mellitus and pre-diabetes in men and women: Evidence from a longitudinal community cohort**

**Supplementary Table 1: Baseline characteristics of participants at baseline**

|  | Overall (n=14,871) | Men (n=7,401) | Women (n=7,380) |
| --- | --- | --- | --- |
| Age (mean (SD)) | 54.98 (11.10) | 55.22 (11.11) | 54.75 (11.09) |
| Women (%) | 7380 (49.6) |  |  |
| SES (mean (SD)) | 12.90 (4.48) | 13.62 (4.62) | 12.17 (4.21) |
| PHQ-9 (mean (SD)) | 4.08 (3.55) | 3.64 (3.38) | 4.53 (3.67) |
| Current depression, PHQ-9≥10 (%) | 1124 (7.7) | 445 (6.1) | 679 (9.4) |
| Depression status^*^ |  |  |  |
| No depression (%) | 12242 (83.9) | 6477 (88.3) | 5765 (79.5) |
| HD (%) | 1221 (8.4) | 415 (5.7) | 806 (11.1) |
| CD (%) | 595 (4.1) | 269 (3.7) | 326 (4.5) |
| PD (%) | 525 (3.6) | 174 (2.4) | 351 (4.8) |
| BMI (mean (SD)) | 27.36 (5.01) | 27.85 (4.29) | 26.86 (5.61) |
| Packyears of smoking (mean (SD)) | 4.87 (11.35) | 5.99 (12.93) | 3.79 (9.45) |
| Diabetes*^†^* | 1260 (8.5) | 772 (10.3) | 488 (6.7) |
| Prediabetes*^‡^* (%) | 4127 (27.9) | 2062 (27.6) | 2065 (28.1) |
| HbA1c (mean (SD)) | 5.56 (0.66) | 5.59 (0.70) | 5.52 (0.62) |
| CRP (mean (SD)) | 2.95 (5.74) | 2.82 (6.14) | 3.09 (5.31) |
| Antidepressant with weight effect (%) | 242 (1.6) | 70 (0.9) | 172 (2.3) |
| Weight-neutral antidepressant (%) | 468 (3.1) | 156 (2.1) | 312 (4.2) |
| Diabetes*^†^* at F/U (%) | 1242 (10.1) | 780 (12.4) | 462 (7.7) |
| Prediabetes*^‡^* at F/U (%) | 3865 (31.4) | 1883 (30.0) | 1982 (33.0) |

*^*^ Classification of depression status: current depression is defined by PHQ-9≥10, prior depression is based on patient’s report of a prior diagnosis of depression (history of depression)*

*^†^ diabetes defined by intake of antidiabetic medication and/or previous diagnosis of type 2 diabetes mellitus and/or HbA1c ≥6.5%.*

*^‡^ Pre-diabetes defined by HbA1c 5.7%–6.4% (following the American Diabetes Association criteria)*

*Abbreviations: SES = socioeconomic status, PHQ-9 = Patient Health Questionnaire – Depression Module, HD = history of depression (without current depression as defined by PHQ-9≥19), CD = current depression (PHQ-9≥10, without history of depression), PD = persistent depression (history of depression and current depression as defined by PHQ-9≥10), BMI = Body Mass Index, HbA1c = Glycated hemoglobin concentration, CRP = C-Reactive Protein*

**Supplementary Table 2: Baseline characteristics of participants without prediabetes and diabetes at baseline**

|  | Overall (n=8,037) | Men (n=4,019) | Women (n=4,018) |
| --- | --- | --- | --- |
| Age (mean (SD)) | 51.74 (10.58) | 52.13 (10.76) | 51.34 (10.38) |
| Women (%) | 4018 (50.0) |  |  |
| SES (mean (SD)) | 13.75 (4.34) | 14.49 (4.46) | 13.01 (4.09) |
| PHQ-9 (mean (SD)) | 3.97 (3.44) | 3.52 (3.24) | 4.41 (3.57) |
| Current depression, PHQ-9≥10 (%) | 551 (6.9) | 210 (5.3) | 341 (8.6) |
| Depression status^*^ |  |  |  |
| No depression (%) | 6762 (85.0) | 3543 (89.2) | 3219 (80.8) |
| HD (%) | 646 (8.1) | 222 (5.6) | 424 (10.6) |
| CD (%) | 303 (3.8) | 133 (3.3) | 170 (4.3) |
| PD (%) | 245 (3.1) | 76 (1.9) | 169 (4.2) |
| BMI (mean (SD)) | 26.34 (4.31) | 27.05 (3.72) | 25.62 (4.72) |
| Packyears of smoking (mean (SD)) | 3.65 (9.26) | 4.10 (10.06) | 3.23 (8.41) |
| HbA1c (mean (SD)) | 5.22 (0.33) | 5.23 (0.32) | 5.21 (0.34) |
| CRP (mean (SD)) | 2.35 (4.12) | 2.16 (3.61) | 2.54 (4.57) |
| Antidepressant with weight effect (%) | 98 (1.2) | 30 (0.7) | 68 (1.7) |
| Weight-neutral antidepressant (%) | 209 (2.6) | 74 (1.8) | 135 (3.4) |
| Diabetes*^†^* at F/U (%) | 96 (1.2) | 58 (1.4) | 38 (1.0) |
| Prediabetes*^‡^* at F/U (%) | 1600 (20.0) | 769 (19.2) | 831 (20.8) |

*^*^ Classification of depression status: current depression is defined by PHQ-9≥10, prior depression is based on patient’s report of a prior diagnosis of depression (history of depression)*

*^†^ diabetes defined by intake of antidiabetic medication and/or previous diagnosis of type 2 diabetes mellitus and/or HbA1c ≥6.5%.*

*^‡^ Pre-diabetes defined by HbA1c 5.7%–6.4% (following the American Diabetes Association criteria)*

*Abbreviations: SES = socioeconomic status, PHQ-9 = Patient Health Questionnaire – Depression Module, HD = history of depression (without current depression as defined by PHQ-9≥19), CD = current depression (PHQ-9≥10, without history of depression), PD = persistent depression (history of depression and current depression as defined by PHQ-9≥10), BMI = Body Mass Index, HbA1c = Glycated hemoglobin concentration, CRP = C-Reactive Protein*

**Supplementary Table 3: Missings (N=14,871 at baseline)**

|  | **Missings** |
| --- | --- |
| Age | 0 |
| Gender | 0 |
| SES | 91 |
| PHQ-9 | 270 |
| BMI | 9 |
| Pack-years | 688 |
| Cholesterol | 25 |
| HDL | 41 |
| LDL | 172 |
| Triglycerides | 28 |
| HbA1c | 57 |
| …at follow-up | 55 |

**Supplementary Table 4: Detailed results of logistic regression analysis: relative odds of incident pre-diabetes and type 2 diabetes mellitus, predicted by depression status (current depression, prior depression, or both) with odds ratios of covariates**

|  | **Prediabetes^*^** | | | |  | **Diabetes^#^** | | | |
| --- | --- | --- | --- | --- | --- | --- | --- | --- | --- |
|  | **OR** | **95%-CI** | | **p-value** |  | **OR** | **95%-CI** | | **p-value** |
|  | *Model 1 (n=7,922)* | | | |  | *Model 1 (n=11,243)* | | | |
| HD | 0.97 | 0.79 | 1.19 | 0.778 |  | 1.04 | 0.71 | 1.47 | 0.848 |
| CD | 1.11 | 0.81 | 1.48 | 0.511 |  | 1.84 | 1.14 | 2.81 | 0.008 |
| PD | 1.19 | 0.86 | 1.61 | 0.276 |  | 2.61 | 1.74 | 3.79 | <0.001 |
| Age | 1.04 | 1.04 | 1.05 | <0.001 |  | 1.04 | 1.03 | 1.05 | <0.001 |
| Gender (Ref.=men) | 1.15 | 1.03 | 1.29 | 0.016 |  | 0.68 | 0.56 | 0.82 | <0.001 |
|  | *Model 2 (n=7,521)* | | | |  | *Model 2 (n=11,203)* | | | |
| HD | 0.95 | 0.77 | 1.16 | 0.628 |  | 1.00 | 0.68 | 1.43 | 0.999 |
| CD | 1.06 | 0.78 | 1.43 | 0.688 |  | 1.79 | 1.11 | 2.74 | 0.011 |
| PD | 1.15 | 0.83 | 1.55 | 0.391 |  | 2.44 | 1.62 | 3.54 | <0.001 |
| Age | 1.04 | 1.03 | 1.05 | <0.001 |  | 1.04 | 1.03 | 1.05 | <0.001 |
| Gender (Ref.=men) | 1.09 | 0.97 | 1.23 | 0.131 |  | 0.6 | 0.49 | 0.74 | <0.001 |
| SES | 0.97 | 0.96 | 0.99 | <0.001 |  | 0.93 | 0.91 | 0.95 | <0.001 |
|  | *Model 3 (n=7,532)* | | | |  | *Model 3 (n=10,706)* | | | |
| HD | 0.85 | 0.67 | 1.06 | 0.152 |  | 0.90 | 0.59 | 1.34 | 0.627 |
| CD | 0.96 | 0.70 | 1.31 | 0.819 |  | 1.41 | 0.85 | 2.24 | 0.162 |
| PD | 0.95 | 0.66 | 1.33 | 0.752 |  | 2.02 | 1.25 | 3.14 | 0.003 |
| Age | 1.04 | 1.03 | 1.05 | <0.001 |  | 1.04 | 1.03 | 1.05 | <0.001 |
| Gender (Ref.=men) | 1.22 | 1.08 | 1.38 | 0.002 |  | 0.68 | 0.55 | 0.84 | <0.001 |
| SES | 0.99 | 0.98 | 1.00 | 0.146 |  | 0.97 | 0.95 | 0.99 | 0.015 |
| BMI | 1.04 | 1.03 | 1.06 | <0.001 |  | 1.15 | 1.13 | 1.17 | <0.001 |
| Log(CRP) | 1.14 | 1.05 | 1.24 | 0.001 |  | 1.12 | 0.98 | 1.27 | 0.088 |
| Log(Packyears) | 1.09 | 1.04 | 1.14 | <0.001 |  | 1.30 | 1.19 | 1.42 | <0.001 |
| Never smoker (Packyears=0) | 0.89 | 0.78 | 1.01 | 0.062 |  | 1.08 | 0.84 | 1.39 | 0.555 |
| Weight neutral antidepressant | 1.21 | 0.82 | 1.76 | 0.330 |  | 0.77 | 0.41 | 1.37 | 0.390 |
| Antidepressant with weight effect | 1.43 | 0.89 | 2.26 | 0.128 |  | 2.25 | 1.19 | 3.99 | 0.008 |

** pre-diabetes defined by HbA1c 5.7%–6.4% (following the American Diabetes Association criteria)*

*^#^ diabetes defined by intake of antidiabetic medication and/or previous diagnosis of type 2 diabetes mellitus and/or HbA1c ≥6.5%.*

*Abbreviations: OR = Odds Ratio, 95%-CI = 95%-Confidence Interval, HD = history of depression (without current depressive episode as definded by PHQ-9≥19), CD = current depression (PHQ-9≥10, without history of depression), PD = persistent depression (history of depression and current depression as defined by PHQ-9≥10), Ref=Reference category, SES = socioeconomic status, BMI = Body Mass Index, CRP = C-reactive protein*
